# Development of the PICCOTEAM Reference Case for Economic Evaluation of Precision Medicine

**DOI:** 10.34172/ijhpm.8756

**Published:** 2025-09-08

**Authors:** Wenjia Chen, Dimple Butani, Yi Wang, Janet Bouttell, Yah Ru Juang, Paul Scuffham, Janneke P.C. Grutters, Alec Morton, Hwee-Lin Wee, Joanne Ngeow, Vorasuk Shotelersuk, Yue Zhang, Laura Huey Mien Lim, Yot Teerawattananon, on behalf of the Working Group

**Affiliations:** ^1^Saw Swee Hock School of Public Health, National University of Singapore, Singapore, Singapore.; ^2^Health Intervention and Technology Assessment Program (HITAP), Ministry of Public Health, Bangkok, Thailand.; ^3^Centre for Healthcare Equipment and Technology Adoption, Nottingham University Hospitals NHS Trust, City Hospital, Nottingham, UK.; ^4^Health Economics and Health Technology Assessment, School of Health and Wellbeing, University of Glasgow, Glasgow, UK.; ^5^Centre for Applied Health Economics, School of Medicine and Dentistry, Griffith University, Nathan, QLD, Australia.; ^6^Menzies Health Institute Queensland, Griffith University, Nathan, QLD, Australia.; ^7^Science Department IQ Health, Radboud University Medical Center, Nijmegen, The Netherlands.; ^8^Department of Management Science, University of Strathclyde Business School, Glasgow, UK.; ^9^Duke-NUS Medical School, Singapore, Singapore.; ^10^Department of Pharmacy, Faculty of Science, National University of Singapore, Singapore, Singapore.; ^11^Cancer Genetics Service, Division of Medical Oncology, National Cancer Centre Singapore, Singapore, Singapore.; ^12^Lee Kong Chian School of Medicine, Nanyang Technological University, Singapore, Singapore.; ^13^Excellence Center for Genomics and Precision Medicine, King Chulalongkorn Memorial Hospital, the Thai Red Cross Society, Bangkok, Thailand.; ^14^Center of Excellence for Medical Genomics, Department of Pediatrics, Faculty of Medicine, Chulalongkorn University, Bangkok, Thailand.

**Keywords:** Precision Medicine, Genetic Test, Sequencing, Economic Evaluation, Cost Effectiveness, Methodology

## Abstract

**Background:**

Current economic evaluations (EEs) of precision medicine (PM) often adhere to generic reference cases (RCs) which overlook the unique healthcare paradigms of PM. This study aimed to develop an RC to standardize the conduct and reporting of EEs of PM.

**Methods:**

A working group comprising 5 core health economists, 22 PM experts, and research staff from Singapore, Thailand, the Netherlands, UK, and Australia who were actively engaged in EE and clinical PM implementation. The RC development comprised four stages: (1) Expert consultation shaping the RC’s scope and structure across nine domains: Population, Intervention, Comparator, Cost, Outcome, Time, Equity and ethics, Adaptability, and Modelling (ie, "PICCOTEAM" framework); (2) A comprehensive literature review on current PM EE approaches and challenges; (3) Obtaining expert consensus and drafting recommendations; (4) A workshop for RC refinement based on stakeholder feedback on relevance and feasibility. Following an experts’ workshop, consensus was reached to tailor PM recommendations for screening, diagnosis, and pharmacogenomics, market-access, and early EEs.

**Results:**

The PICCOTEAM RC offers 46 recommendations for conventional EEs to guide PM reimbursement, emphasizing expert engagement, iterative study processes, disease-specific outcomes, decision uncertainty analyses, and equity considerations. Additionally, 30 recommendations are provided for early-stage evaluation to enhance PM’s positioning and value proposition, mitigating uncertainty, equity, and ethical issues.

**Conclusion:**

The PICCOTEAM RC offers a standardized process to conduct and report diverse PM EEs. This will serve as guidance for health departments, researchers, clinicians, editors, and reviewers. Pilot testing and continuous updates are recommended for ongoing relevance and applicability of this RC.

## Background

**Key Messages**

**Implications for policy makers**

- Standardized and adaptable economic evaluations (EEs): Despite various generic health technology assessment (HTA) guidelines and tools, evaluating precision medicine (PM) remains challenging due to its unique healthcare paradigm, complex evidence, and ethical concerns. The PICCOTEAM reference case (RC) standardizes and strengthens PM EE across both early development and market-access stages, ensuring high-quality, comparable, and adaptable value-for-money assessments in diverse healthcare settings.
- Support for resource-limited settings: Policy-makers in low- and middle-income countries (LMICs) often face research capacity constraints, making it challenging to evaluate complex technologies even with generic tools. The RC offers structured guidance to streamline assessments, reduce reliance on extensive expert input, and support timely PM adoption. By enhancing efficiency and accessibility, the RC empowers diverse healthcare systems to make well-informed decisions on PM implementation.
- Improved transferability of evidence: The RC complements generic frameworks but functions as a practical, operational manual tailored to the unique challenges of PM, particularly in diverse, resource-constrained settings. Its emphasis on transparency, consistency, and bias minimization helps LMICs overcome data and expertise gaps. Policy-makers can leverage EEs from similar settings that adhere to the RC recommendations, thereby reducing the burden of independent assessments.

**Implications for the public**

The PICCOTEAM reference case (RC) offers a structured yet flexible framework for evaluating a wide range of PM strategies, ensuring consistent, high-quality, and adaptable evidence generation across various stages of the development lifecycle. By supporting informed decision-making and efficient resource allocation, it enables the prioritization of high-value, cost-effective, and sustainable PM technologies that can benefit both present and future generations. Moreover, the RC acts as a collaborative platform to guide unbiased early EEs, reduce implementation risks, and optimize PM development. It also encourages and fosters meaningful stakeholder engagement among developers, clinicians, health economists, and patient advocates for effective PM development and implementation.

Precision medicine (PM) is a novel medical approach that tailors clinical interventions based on gene profiling results,^1,2^ such as genetic testing to screen for disease risk factors (eg, *BRCA1/2* testing for hereditary breast and ovarian cancers), diagnosis of genetic disease (eg, pediatric genetic diseases),^3,4^ and pharmacogenomic testing for drug responses and adverse reactions.^5^ Cascade testing, screening relatives of mutation carriers, is essential in facilitating early detection and tailored interventions amid budget constraints, thereby enhancing PM’s efficiency.^6^ Introducing new PM approaches into healthcare systems requires a thorough health technology assessment (HTA).^7^ Early-stage HTA, spanning from conceptualization to first clinical use, informs innovators about the potential roles of a developing technology in clinical pathways, its target product profiles, design and evidence generation strategy and its reimbursement prospects at market access.^8^ Conventional HTA evaluates health technologies at market access stage and beyond to help regulators, payers, and patients make decisions about regulation, reimbursement, clinical guidelines development, and technology use. Economic evaluation (EE), a key component of HTA, assesses new technologies against alternatives using cost-effectiveness, cost-utility, and cost-benefit analyses to inform decision-making.

 To ensure transparency, consistency, and to minimize bias in EE, “reference cases (RCs)” are often used to streamline and standardize the planning, conducting, reporting, and appraising processes.^9-11^ In 2022, our research team from the Saw Swee Hock School of Public Health, National University of Singapore (NUS-SSHSPH), Health Intervention and Technology Assessment Program (HITAP) (Thai Ministry of Public Health), and Precision Health Research, Singapore (PRECISE) conducted systematic reviews of cost-utility analyses of PM^12^ and the methodological challenges in PM EE.^13^ These reviews highlighted key issues in PM EE: underrepresentation of low- and middle-income countries (LMICs), systematic study bias, and the limitations of generic RCs in addressing PM’s unique healthcare paradigms.^12,14^ The 2025 *HTA International, HTAsiaLink and ISPOR (International Society for Pharmacoeconomics and Outcomes Research) Special Task Force *offers best practice guidance for HTA guidelines, particularly in LMICs, which proposed guideline development frameworks and summarized key HTA resources, checklists, and guidance for different aspects of evaluation (eg, clinical assessment, modeling, uncertainty analysis, reporting) and even evaluations of diagnostic tests and complex technologies.^15^ However, most guidelines are generic, focusing on single “cure-based” interventions in (hypothetically) homogeneous populations,^9-11^ whereas checklists and guidance primarily focus on discrete aspects of evidence^16^ or to standardize reporting.^17^ In contrast, PM evaluation encounters unique challenges amid rapid technological advancements, resulting in an increasingly complex and uncertain clinical decision space, such as next generation sequencing and other omic-based technologies. Meanwhile, medical diagnostics and technology evaluations are fragmented across HTA and reimbursement structures^18-21^; those structures are insufficient for studying PM in multi-gene contexts or as evolving test-treatment hybrids. Moreover, PM is often applied in populations or subgroups with racially diverse genetic backgrounds, where genetic influences may be lifetime or intergenerational, whereas evidence from small, stratified sub-populations is difficult to obtain and lagged, often due to delayed data collection, analysis, and representation in research.^13^ Notably, clinical adoption of PM frequently also encounters unequal ethnicity-related uptake and patient confidentiality concerns in cascade testing.^13^ Although PM can be assessed using a generic complex technology model, such as INTEGRATE-HTA,^22^ the critical steps of creating a logic model, defining architecture and identifying specific attributes using local insights remain particularly challenging in resource-constrained countries.

 To overcome those challenges, a new, flexible RC tailored to diverse decision-making contexts across various PM types and stages can empower institutions, policy-makers, and clinicians to utilize economic evidence effectively while considering its limitations, adaptability, and ethical and equity concerns. While generic guidelines and tools enclosed in the ISPOR Best Practice Task Force may serve as valuable references or an “encyclopedia” when needed, the PM-specific RC will function as an operational manual, offering hands-on guidance to navigate PM-specific challenges, in particular for researchers and settings with limited capacity. By enhancing the transferability of PM EEs across settings, the RC can potentially reduce the burden to LMICs of conducting independent assessments. Additionally, integrating early EE into the RC will help support the cost-effective development and implementation of PM technologies.

 This study aimed to develop this new RC, hereafter referred to as the “PICCOTEAM Reference Case,”to support evidence-informed decision-making by guiding Early EE for PM development and conventional EE for reimbursement, tailored to different PM intervention types. Different from national reimbursement guidelines (eg, the National Institute for Health and Care Excellence)^9^ or reporting checklists (eg, Consolidated Health Economic Evaluation Reporting Standards),^17^ this RC sought to offer end-to-end methodological guidance to comprehensively and specifically address the unique complexities of PM. The PICCOTEAM RC was developed with input from two reviews,^12,13^ and expert opinion from the research team, which has conducted EEs to inform coverage decisions for the governments in Asia, the Americas, Africa, and Europe for decades. Furthermore, the RC has been evaluated for relevance and feasibility in 5 national clinical implementation pilot studies in Singapore and 2 in Thailand.

## Methods

 The PICCOTEAM RC followed a structured four-stage approach, adapted from health research reporting guidelines.^23^ Figure illustrates the study process.

**Figure:**
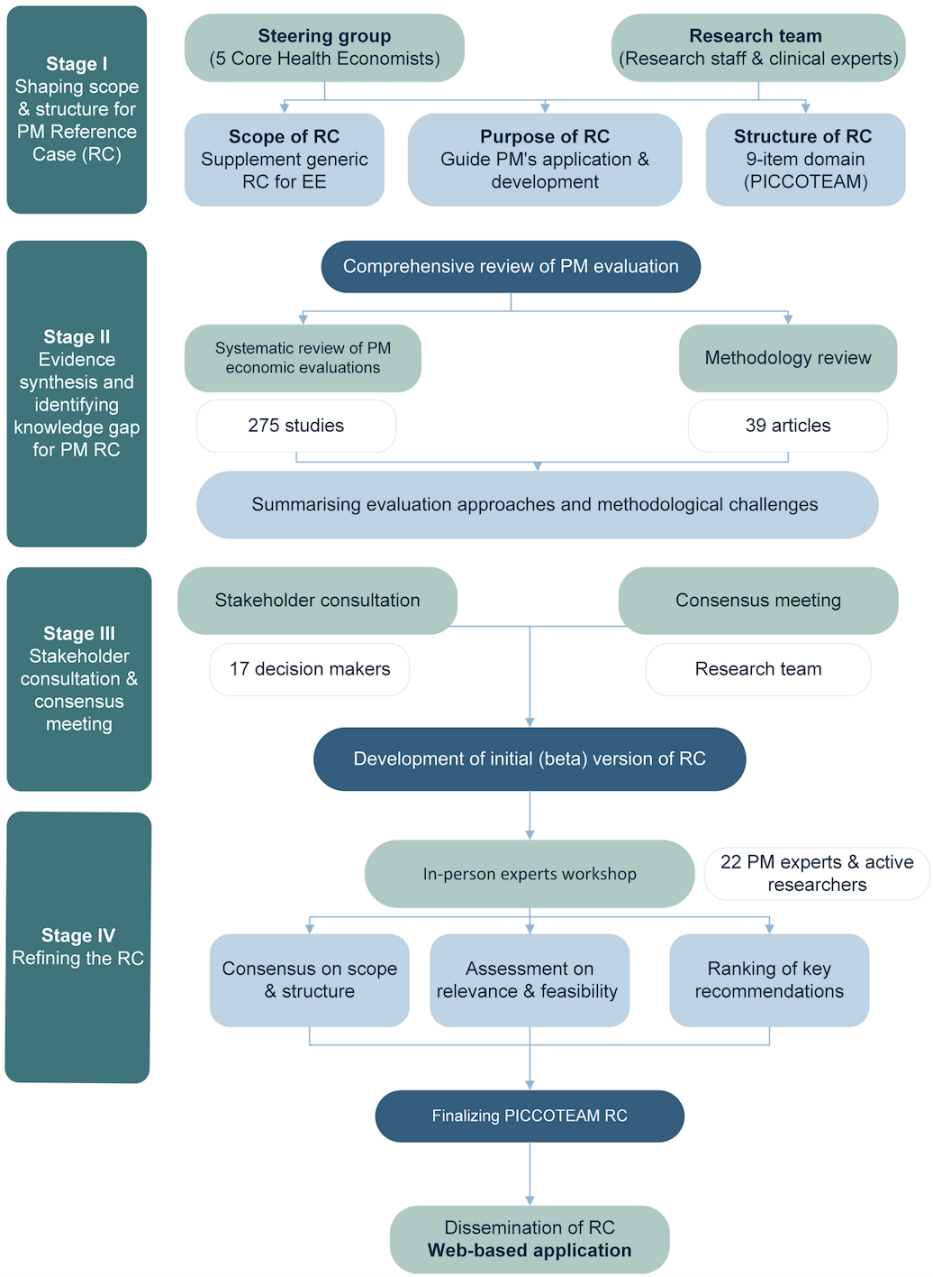
A Schematic Diagram of Developing the PICCOTEAM Reference Case. Abbreviations: PM, precision medicine; EE, economic evaluation.

###  Stage 1. Scope and Structure 

 First, a working group was formed, constituting a steering committee of 5 experts (CWJ, WY, YT, AM, and PS) in EE and PM research from NUS-SSHSPH, Singapore; HITAP, Thailand and Griffith University, Australia, alongside research assistants and associates, doctoral and post-doctoral trainees from the National University of Singapore, HITAP, and external clinical consultants specializing in health economics and outcomes research and/or PM. The working group defined the scope of this PM-RC, which would supplement generic RCs for guiding original PM EE studies. This RC aimed to clarify considerations for EE in PM applications (screening, diagnostic, pharmacogenomic) and clinical stages (conventional or early EEs), guiding design, conduct and reporting for unbiased and comparable estimates of PM’s value for money. Additionally, the RC aimed to address prominent issues in inferring PM-relevant recommendations alongside the EE, particularly ethics and equity concerns, and adaptability of evidence to other settings. Finally, the working group determined the structure of RC, consisting of 9 domains: Patient population, Intervention, Comparator, Cost, Outcome, Time, Equity and ethics, Adaptability, and Modelling aspects, namely the “PICCOTEAM” framework (Table 1).

**Table 1:** The PICCOTEAM Reference Case for Conventional Economic Evaluation of Precision Medicine

	**RC Recommendations **
Population	Defining the target population (either the full population without a genetic diagnosis^*^ or stratified with known genetic information). ^*^ Screening and pharmacogenomic test: consider genetic ancestry background (eg, admixture measurement^a,24^).
	The scope of target population is specific to the intervention type. Screening test: Consider the entire population suitable for screening where feasible. Diagnostic test: Focus on probands and their relatives and include their potential cost/benefits. Pharmacogenomic test: Consider all relevant patient groups for the treatment.
	Update and expand the target population definition as PM test evolves along the clinical pathway.
Intervention	Define intervention scenarios relevant to the setting and practical for the decision-makers, considering multiple and/or overlapping applications: (i) Screening/monitoring PM: Among unaffected population, stratify risk for genetic conditions (eg, FH/Hereditary breast & ovarian cancers, polygenic risk scores for non-communicable diseases). (ii) Diagnostic PM – early diagnosis: Early disease diagnosis, eg, paediatric genetic disease, rare diseases. (iii) Diagnostic PM – genetic subtype: Among affected population, diagnose genetic subtypes with differential risks of adverse outcomes and/or progression, for treatment stratification. (iv) Pharmacogenomic PM: Predict adverse drug reaction, treatment efficacy and/or drug dosage to stratify patients to optimize treatment, eg, HLA-B*1502 test. Specify drug options, country and time of application.
	Define the intervention pathway: First, explore clinical pathways using a systematic framework across five domains (ie, test delivery, result, diagnostic and treatment decisions, and implementation), considering timing, feasibility, accuracy, decision uncertainty, treatment efficacy, and adherence; Next, determine main intervention strategies based on relevance and feasibility; others can serve as comparators. Discuss intervention characteristics (test delivery, accuracy and result, diagnostic and treatment decisions, uncertainty, implementation and adherence), timing and feasibility.
Comparator	Follow generic guidelines to consider current standard of care as a common comparator.
	Provide justification for choice of comparators.
	Consider clinical expert elicitation, consider all possible comparators given the feasibility, both genomic and non-genomic options, and subsequent therapeutic decisions. Include policy discussion if possible.
Costs and outcomes (1) - Costs	Measure costs from a societal perspective or compare multiple perspectives if the societal viewpoint is not preferred locally.
	Identify all relevant direct medical costs including patient recruitment (eg, education, counsellors, outreach to relatives), sample collection and testing (including cascade testing), result interpretation, communication and actions taken based on results, monitoring disease progression and drug response, and infrastructure (eg, data storage) etc.
	Adopt expert opinion to determine relevant and potentially influential cost items to be measured and distinguish costs that directly impact cost-effectiveness from budget impact.
	Consider and/or discuss the impact of variations in PM unit costs across facilities, countries and time due to economies of scope (cost advantages experienced when services/products variety increases) and economies of scale (cost advantages experienced when production becomes efficient), and intangible costs (eg, diagnostic odyssey, children’s education, caregiver burden).
	Consider full costs, including PM tests, even if they are subsidised by manufacturer, unless it is not aligned with the study perspective.
	Consider cost differentiation between positive and negative test results, and cost differentiation between different turnaround time for test results.
Costs and outcomes (2) - outcomes	Include a societal and/or compare multiple perspectives, follow common guidelines and disease models to identify disease-specific outcomes (especially core outcome sets from effectiveness trials),^25,26^ and/or symptom improvements, consider harms and social values (eg, social prescribing and personalization); justify outcome choice.
	Evidence selection and adjustment: (i) In PM, follow guidelines and hierarchy of evidence for extrapolating treatment effect (eg, use single-arm trials if good quality historical controls are available, the natural disease history is well known, and population is homogenous).
	(ii) To extrapolate real-world evidence, select observational studies which applied statistical methods to estimate causality via controlling for the risks of selection bias and confounding where feasible.
	Select the appropriate outcome(s) in the evaluation and justify the choice: (i) Disease specific health-related outcome or general health-related outcomes.
	(ii) Impact on labour force participation (eg, human capital, work productivity), social value (including intergenerational impact) should be included where relevant, feasible, or discussed.
	Discuss the impact of PM interventions on other family members including effects on their physical or mental health, as well as their financial well-being where feasible.
	When surrogate outcomes are used, justify and perform validation on: (i) Biological plausibility.
	(ii) The association between surrogate and final outcome, as well as the effect on technology on both surrogate and final outcomes, across cohorts or at the level of the individual patient, perform individual-level analysis where feasible.
Costs and Outcomes (3) - Bias control	Assess risk of bias of PM test accuracy using standardized tools (eg, QUADAS-2).^27^
	Assess clinical relevance of risk classification using clinical utility analysis, such as Decision Curve Analysis,^28^ to identify meaningful risk thresholds and ensure net benefit.
	Assess risk of bias of real-world data (costs & outcomes) using standardized tools (eg, ROBINS-I^29^).
	In the absence of data, use an RC^30^ or structured expert elicitation method^31^ to obtain outcomes or surrogates.
	Ensure consistency in data sources and parameters of the same evidence type across risk-stratified subgroups to minimize bias due to data heterogeneity.
	Clearly explain and justify, with caution and transparency, any inclusion or exclusion of evidence that could be biased.
Time horizon	Use an extended time horizon to capture lifetime and intergenerational advantages when modelling costs and effects in PM tests used for screening and diagnosis.
	Discount future costs and outcomes based on most recent national guidelines using discounting rate comparable to EEs for non-PM interventions.
	Apply the Social-Welfare-Equivalent discount rate^b,32^ when estimating intergenerational impact.
Equity and Ethics	Ensure principles of non-discrimination and equality of opportunity are applied in the analysis and policy recommendations.
	For relevant topics, incorporate health inequality into the EE wherever applicable, eg, distributional cost-effectiveness analysis^c,33^ or similar approaches.
Adaptability	To enhance finding generalisability, use a generic disease model, nationally representative data and multiple scenarios analyses wherever applicable.
Modelling issues	Use a societal and/or consider multiple analytical perspectives in settings where the societal perspective is not the preferred option.
	Include an “impact inventory,” ie, a structured table listing the health and non–health-related effects of an intervention.
	Adhere to methodological guideline recommendations, eg, ISPOR Modelling Good Research Practice.^34^
	Leverage expert opinions to define model structure: Begin with pre-specified intervention and comparator pathways, and combining test sensitivity and specificity wherever applicable.
	Incorporate test sensitivity, specificity, and adverse effects and disutility from harm, and consider key epidemiological data for determining PM performance of all policy options.
	Model selection should follow best practice guidelines, using approaches like patient-level state transition microsimulation, or discrete event simulation for complex structures and high uncertainty.
	Data extrapolation: Follow best practice guidelines and landmark models (eg, mixture cure^d,35^ and response-based models^e,36^) to extrapolate lifetime/intergenerational costs and benefits; Use causal inference to address bias and confounding in observational studies.
	Routine bias assessments^37^ at key stages such as model design, evidence selection, parameterization.
	Conduct model validation such as internal validity, face validity (eg, model structure, data sources, assumptions, and results), predictive and cross-validity, and identify unvalidatable parts specific to PM.
	Consider structural uncertainty in sensitivity analysis by adding uncertain parameters to represent model scenario choices (eg, uncertainty around PM uptake and compliance of clinicians and patients); Develop evidence-based decision support tools and patient decision aids for knowledge translation.
	Present results from budget impact analysis that is relevant to decision-making in a given jurisdiction.
	Breakdown the impact of PM intervention on societal and healthcare resource use (eg, hospital services and manpower), to support infrastructure setup and/or prioritize disease indications for evaluation.

Abbreviations: EE, economic evaluation; FH, familial hypercholesterolemia; ISPOR, International Society for Pharmacoeconomics and Outcomes Research; PM, precision medicine; QUADAS, Quality Assessment of Diagnostic Accuracy Studies; RC, reference case; ROBINS-I, Risk Of Bias In Non-randomised Studies - of Interventions. ^a^Admixture analysis refers to the quantitative assessment of ancestral genetic contributions from multiple parental populations within individuals or populations. ^b^Social-Welfare-Equivalent discount rate calculates the present value of costs and benefits associated with healthcare interventions, considering the societal perspective and welfare implications. ^c^Distributional cost-effectiveness analysis is an analytical approach used to assess the distributional impact of healthcare interventions on health outcomes and costs across different population subgroups, considering equity concerns. ^d^Mixture cure models are statistical models used to analyze survival data when there are two subpopulations with distinct survival mechanisms: one that is cured (ie, does not experience the event of interest) and one that is susceptible to the event. ^e^Response-based models are statistical models used to analyze longitudinal data where the response variable may change over time due to various factors and account for correlations between repeated measurements within individuals.

###  Stage 2. Evidence Synthesis From Previous Reviews

 To shape this RC’s content, we performed a systematic review on PM EEs published between 2011 and 2021, alongside a targeted review of methodological and policy challenges in performing PM EEs during this period. The results were published elsewhere.^12,13^ We reviewed 5187 papers on PM’s cost-effectiveness published from 2011 to 2021, as earlier PM research was considered less relevant to current clinical practice. After a systematic review, we found 275 relevant papers. We explored drivers of heterogeneity in PM’s cost-effectiveness, assessed study and publication biases,and summarized common design approaches based on clinical stages using the PICCOTEAM framework.^12^ Next, we reviewed 39 methodology papers for performing PM EEs, stratified by PM types and clinical stages. We then summarized common methodological and policy challenges of PM EEs, alongside recommendations for good practice, leading to the development of the PICCOTEAM framework.^13^ Results of these reviews were then synthesized into the initial RC draft.

###  Stage 3. Stakeholder Consultation and Expert Consensus

 We surveyed 17 stakeholders from relevant decision-making bodies and HTA agencies in Singapore and Thailand during a workshop in February 2022, and ranked the importance of EE components affecting PM-related decision-making, with results published.^13^ Afterwards, an internal virtual consensus meeting with co-authors was conducted in June 2023. Feedback and agreement on the RC’s scope, structure, and content were collected and integrated into the beta-version of this RC for piloting.

###  Stage 4. Refining the Reference Case 

 A three-day PM RC workshop convened in Khao Yai, Thailand, in June 2023, sought stakeholder input on the RC’s relevance, feasibility, importance, and potential oversights for piloting and refinement. Detailed process and findings from this workshop are being documented in a separate workshop paper. Participants constituted a multidisciplinary group (n = 22) including oncologists, geneticists, clinical researchers, health economists, and public health agency with expertise in PM from both Singapore and Thailand. Less than half (40%) of this group participated in the first workshop. In this workshop, we collected participant feedback via a survey using a standardized approach. First, participants were asked to agree or disagree on the overall scope and structure of RC, followed by rating the relevance and feasibility of each recommended item in the PICCOTEAM domains. A 1-to-5 Likert scale was used for rating, with 1 indicating irrelevance/not feasible and 5 indicating high relevance/feasibility. Next, statistical analyses were performed to summarize the characteristics of workshop participants, and calculate the expected value of relevance and feasibility scores for each recommendation item, using the formula: *E*(*X*) = ∑*xP*(*x*), where E(X) represents the expected value, x denotes the possible scores, and P(x) is the probability of each score occurring. This method appropriately accounted for variations in expert opinions and mitigate the influence of extreme responses. Any item with an expected Likert score for either relevance or feasibility ≤3 (ie, neutral to not recommended) was removed from the RC. Participants also rated the importance of recommended items within the nine PICCOTEAM domains on a 1-to-5 Likert scale. We calculated the expected value of these scores to rank the items by their importance. Feedback on missing aspects, revisions, solutions, and supporting studies was also integrated into the revised RC. Finally, we invited key opinion leaders, including 2 clinical experts, 3 health economists, and 2 early HTA experts to review, and finalize the RC’s content, structure, and wording.

###  Dissemination of PICCOTEAM RC Into a Freely Accessible Web-Based Application

 The finalized RC was integrated into a free-accessible web-based application: https://piccoteam.gear4health.com/, developed on the R Shiny interface, featuring user-friendly elements like branching logic, and piping. This web application enables customized recommendations aligning with chosen types of PM application and clinical stage. Users are encouraged to comment on the feasibility of each recommendation, and/or report challenges preventing implementation. These comments will be analyzed and updated in the future.

## Results

###  How to Use the PICCOTEAM RC 

 The PICCOTEAM RC offers tailored support for conducting and reporting PM EEs, adaptable to various application types and developmental stages. Potential users encompass health departments like Ministries of Health, HTA bodies and medical innovators critical for decision-making in PM. This resource is also invaluable to researchers, clinicians, journal editors, manuscript reviewers, and readers evaluating PM economic assessments. The PICCOTEAM RC has been integrated into a freely accessible, user-friendly web app (https://piccoteam.gear4health.com/), enabling users to generate customized recommendation reports. Researchers first select the developmental stage—either Early EE for PM development or Conventional EE for reimbursement and clinical guidelines, followed by the PM type under evaluation.

 In a case study, we have applied the PICCOTEAM RC to assess the cost-effectiveness of implementing whole exome sequencing for cascade testing of familial hypercholesterolemia (FH) in Thailand. In parallel, we used this RC to guide an Early EE to evaluate the target cost-performance profile for developing long-read sequencing, to determine whether long-read sequencing could be prioritized over existing methods for FH cascade testing in Thailand. The findings will be published in a separate paper.

###  Recommendations for Conventional EE of PM 

Table 1 outlines 46 recommendations for conducting conventional EE on PM across 9 key clinical and economic domains, adapted to three genetic test types: (1) screening test for disease risk stratification based on genetic predispositions (eg, hereditary breast and ovarian cancers), (2) diagnostic test for early diagnosis of undetected disease or diagnosis of genetic subtypes among affected population, and (3) pharmacogenomic test for prediction adverse drug reactions, treatment efficacy and/or optimized drug dosage (eg, HLA-B*1502 test).

####  Population

 Due to the evolving patient categorization, expanding target groups like cascade testing, and inherent heterogeneity from genetic subgroups, defining a target population is challenging in PM evaluation.^13^

 Recommendation: The scope of target population should be specific to the type of PM testing. The PICCOTEAM RC recommends defining the target population composition, consider whether it is total and unstratified (ie, targeting everyone without any specific categories, eg, population-based genetic screening and/or pharmacogenomic testing), or already stratified and affected (ie, already divided into groups based on certain characteristics, eg, confirmatory testing, cascade testing, targeted therapy). If the target population is unstratified, racial genetic background should be considered. For instance, the prevalence estimates for pathogenic genotype variants should reflect the racial/ethnic makeup of the population. Of note, as the PM test evolves, target population’s definition should be updated accordingly.

####  Intervention and Comparator 

 The complex and evolving clinical decision space of PM makes it challenging to adopt the traditional comparative effectiveness paradigms like the average treatment effect.^13^

 Recommendations: Consultation with clinical experts is recommended to define intervention pathways and select comparators. A systematic approach to explore the full decision space of PM interventions,^38^ from testing to treatment, is advised as an initial step. This follows identifying all possible intervention pathways, assessing decision uncertainty in the pathways, and critically appraising their relevance and feasibility to determine main intervention strategies. During the process, all possible comparators, both PM and non-PM, should be considered and shortlisted, with the current standard of care recommended to be the common comparator.

####  Costs and Outcomes

*Costs:* PM costs vary due to factors including laboratory differences, test results, economies of scale and scope. Often overlooked are manufacturer costs, genetic counselling, patient outreach, and related procedures.^13^

 Recommendations: (1) From a societal perspective and/or multiple perspectives if the societal viewpoint is not preferred locally, use expert opinion to draft a prioritized checklist of relevant cost items, highlighting items that more directly impact cost-effectiveness rather than the budget. (2) During this process, consider the comprehensive scope of direct medical cost – from patient recruitment, sample collection, testing, to test delivery, follow-up actions, disease progression and adverse events. (3) Consider cost variation due to PM results and turnaround time (eg, sequential testing of test positives and negatives), settings, economies of scale (ie, cost advantages due to a larger scale of production of the same product and/or increased throughput on different sequencing platforms), economies of scope (ie, decreases in production cost due to an increasing variety of goods produced), and discuss their potential impacts.

*Outcomes:* Real-world evidence lacks focus on PM’s direct impacts, long-term interventional effects, and data quality, whereas randomized controlled trials struggle with individualized effects within PM’s complex subgroups.^13^

 Recommendations: (1) To navigate PM’s evolving scope and complexity, prioritize a “disease-oriented” evaluation strategy, emphasizing disease-specific outcomes from guidelines and reference disease models, particularly the core outcome sets from effectiveness trials.^25,26^ (2) Follow clinical evidence’s hierarchy for extrapolation of treatment effects.^39,40^ (3) When extrapolating real-world evidence, select observational studies which mitigated selection bias and confounding through robust statistical methods.^41^ (4) Consider a societal perspective and/or multiple perspectives if societal viewpoint is not prioritized in the jurisdiction. For instance, justify outcome selection and discuss PM’s impacts on social value, and family members. (5) When evidence is lacking or a limited budget is available, use validated surrogate outcomes with biological plausibility, proven association with final outcomes, and demonstrated clinical effectiveness on both surrogates and final outcomes.

*Risk of bias:* A unique challenge in PM evaluation is the scarcity of robust real-world evidence. As data is stratified into ever-smaller subgroups over complex, patient-specific pathways, drawing reliable inferences is increasingly difficult, often requiring expert judgment to bridge the gaps.^13^

 Recommendations: (1) Follow established generic tools^15^ to assess risk of bias in PM test accuracy (eg, QUADAS-2^27^), real-world evidence for costs and outcomes (eg, ROBINS-I^29^), and to conduct expert elicitation (eg, RC or structured framework).^30,31^ (2) Ensure the relevance of risk stratification via clinical utility analysis, such as decision curve analysis, which compares potential harms and benefits of tests across reasonable risk threshold ranges and identifies meaningful risk thresholds and patient subgroups for favorable PM use. This method has been widely applied in novel diagnostic context, including genetic testing.^42-44^ (3) Maintain consistency in data sources and parameters across risk-stratified subgroups to minimize bias stemming from evidence heterogeneity, as inconsistent data can obscure true effects and lead to misleading conclusions.

####  Time Horizon

 The temporal aspect is crucial in PM, because one PM test can yield potential lifetime to intergenerational benefits (eg, genetic tests for heritable conditions) whereas the real timeframe of benefit for an individual might be difficult to ascertain due to limited survival data.^13^

 Recommendation: (1) Use an extended time horizon to capture lifetime and intergenerational advantages when modelling costs and effects in PM tests used for screening and diagnosis, this is particularly relevant when employing different time horizons for scenario analysis. (2) While the life-time discount rate of PM costs and outcomes are comparable to non-PM health interventions, consider the social-welfare-equivalent discount rate^32^ for intergenerational impacts as the latter accounts for both time preference for consumption and well-being-related distributional concerns of different generations.

####  Equity and Ethics 

 The rapid commercialization of PM brings potential ethical and equality issues for regulators.^13^

 Recommendation: Incorporate non-discrimination and equality principles in the analysis and policy recommendations, especially for targeted treatment within ancestry and heritage-stratified risk groups, rare disease management, and insurance coverage decisions. If PM can potentially contribute to exacerbations of existing health inequalities, consider health inequality in EEs, particularly for PM screening tests, potentially employing distributional cost-effectiveness analysis.^33^

####  Adaptability

 Adaptability refers to the ability of PM EE to accommodate future changes or adjust to the dynamic and evolving nature of PM interventions.

 Recommendation: As the field of PM relies heavily on ongoing research and the integration of large datasets, the RC recommends using generic disease models and/or utilize nationally representative data and analyzing multiple scenarios and overtime to accommodate the rapidly changing landscape.

####  Modelling

 There is a lack of guidance on modelling PM’s dynamic and complex clinical decision space appropriately, which fosters high uncertainty in modelled clinical pathways and decisions, while common evidence gaps in PM further challenge accurate long-term cost and benefit estimations.^13^

 Recommendation: Expanding from the ISPOR Modelling Good Research Practice,^34^ the PICCOTEAM RC offered 9 additional PM-specific recommendations: (1) Adopt a societal perspective, and/or compare multiple perspectives if the societal viewpoint is not preferred by the jurisdiction. (2) Draft an “impact inventory” which encompasses PM’s health and non-health-related effects. (3) Integrate clinical expert knowledge to shape model structure. (4) Consider PM test accuracy, adverse effects, and harm disutility. (5) Choose a model method aligned with best practices and/or reference studies of that specific type of PM applications. (6) When relevant, follow best practice guidelines and landmark models to extrapolate lifetime to intergenerational costs and benefits of PM from real-world evidence, addressing causal inference. (7) Conduct routine bias assessment^37^ and validate the model, including face validity of model structure, data sources, assumptions and results, identifying unvalidatable parts specific to PM (eg, arbitrary risk threshold, undetected alleles). (8) Parameterize model’s structural uncertainty to enable subsequent sensitivity analysis,^45^ particularly decision uncertainties (eg, uptake rate, risk threshold for treatment escalation) and compliance (eg, physician and patient compliance to PM-guided treatment). (9) To enhance decision-making support, break down PM’s impacts into societal and healthcare aspects, including budget impact analysis results.

###  Recommendations for Early Economic Evaluation of Precision Medicine 

 Currently, early EEs of PM follow similar approaches as the conventional EEs, which may not be insufficient for early-cycle decision-making. Table 2 presents 30 specific recommendations for early EE on PM within the same PICCOTEAM framework.^13^

**Table 2:** The PICCOTEAM Reference Case for Early-Stage Economic Evaluation of Precision Medicine

	**RC Recommendations **
Population	At an earlier stage, envision diverse populations and settings for potential use cases, then prioritize use cases and target populations for evidence generation based on technology development, external changes and stakeholder dialogues.
	Once seeking adoption or approved, limit to relevant populations to clarify use cases in either general- or risk-stratified targeted populations.
Intervention	The scope of intervention should be flexible at early stage: Begin with exploring all possible clinical pathways, consider healthcare system readiness and public perception, eliminate dominated or infeasible options during PM development.
	Apply expert elicitation for formative evaluation to inform technology: (i) Development: Assess the required diagnostic performance to inform decision-makers about potential adoption, notably false positives and false negatives of PM tests, patient uptake, and treatment compliance (eg, test-treatment PM).
	(ii) Positioning: Explore different clinical pathways (via scenario analysis^46^) and the specific design/s of the intervention (via deterministic sensitivity analysis, probabilistic sensitivity analysis) to identify the best alternative or a set of reasonable alternatives; Consider the mid-term and long-term plans of the medical innovator when shaping the intervention (eg, development of related technologies in sequences/parallel or plans to continuously develop and expand the current PM).
	(iii) Product profiles & value proposition: Assess all possible benefits of PM intervention: eg, downstream impact of improved accuracy of risk stratification on healthcare provision, therapeutic options and patient outcomes; Explore the capacity constraints, resource needs, and readiness of the current healthcare system in supporting the intervention; Understand the product profiles that can fit the current healthcare system; Use simulation exercises to determine whether cascade outreach is potentially beneficial.
	New options may be integrated during the development of the technology and/or external changes.
Comparator	Choose the primary competitor based on relevance and acceptance for estimating the value of new PM, consider current practice, gold standard, patient preference, competing and emerging technologies, both PM and non-PM. Involve key stakeholders in decision-making.
Costs & outcomes	Prioritize a checklist of to-be-measured costs, outcomes and potential value that informs outcome at early PM development.
	Identify the most valuable information for building a case for comparative effectiveness and value, and if relevant, use robust expert elicitation approach.
	Consider surrogate or short-term outcomes when faced with insufficient data, using expert elicitation and/or threshold analyses to determine the optimal combinations of price and performance.
	Update cost information timely, explore the budget impacts if needed (implementation, training, recruitment & cascade outreach & counselling, testing & analysis, actions following test rests, disease monitoring) on family and society, and downstream impact on cost-effectiveness.
	Factor in incidental findings and harmful outcomes if possible.
	Use expert elicitation and discussions with developers to estimate the cost of the intervention, explore alternative costs using scenario analysis, assess relevant societal values when feasible.
	Apply an RC^30^ or structured framework^31^ for expert elicitation.
Time horizon	Tailor the time horizon to decision-maker’s perspective, PM technology type and developmental stage.
	The ideal would be to consider lifetime to intergenerational impacts if appropriate; Otherwise, a more pragmatic approach is to consider a shorter time horizon to assess imminent to short-term impacts, in particular for rapidly evolving PM technology.
	Engage expert knowledge to determine the acceptable minimal time horizon and the ideal time horizon.
	Integrate appropriate time horizon into the data collection plan.
Equity and ethics	Apply multidisciplinary stakeholder consultation, including the end users, to explore equity and ethical issues at an early stage.
	Evaluate the impacts of conducting early EE and/or value of information analyses on the equity and ethical considerations of both PM products and the research of PM products.
Adaptability	Initiate early and routine conversations with regulatory agencies, clinicians, patients, and payers to learn their perspectives and needs, aligning with current and future regulatory standards and reimbursement policies.
	Clarify the key factors that affect the applicability of the proposed PM intervention in other settings.
Modelling issues	Use formative, adaptable, and exploratory modelling.
	Start with relatively simple and flexible methods at earlier stage to provide a rapid indication of potential value, eg, scenario analysis^a,46^ and sensitivity analysis.
	Increase the depth of analysis in later stages as more information becomes available and attention is centred on fewer diagnostic strategies and more focused research, eg, value of information analysis.
	Based on the aims and development stage, choose a "headroom" method^b,47^ to identify what innovation has to achieve to be cost-effective across a range of value threshold, expected costs and scenarios, and/or (later) conduct stochastic analysis^c,48^ to consider all parameter uncertainty in the interventions and comparators in early EE models.
	Conduct uncertainty analysis to identify robust intervention strategies.
	Use Value of Information methods,^49,50^ especially Expected Value of Partial Perfect Information, when data are insufficient to identify and prioritize research on key uncertainties.
	Interactive collaboration between stakeholder interviews and decision modelling to enable iterative analyses and assessing the potential value of PM beyond cost-effectiveness metrics.

Abbreviations: EE, economic evaluation; PM, precision medicine; RC, reference case. ^a^Scenario analysis refers to a technique used to systematically explore the potential impact of different assumptions, variables, or scenarios on the outcomes of a health economic model. ^b^The “headroom” method determines the maximum additional cost a new intervention can incur while still being considered cost-effective, aiding in identifying thresholds for incremental cost-effectiveness ratio beyond which the intervention may not be cost-effective. ^c^Stochastic analysis involves incorporating randomness or uncertainty into models through probability distributions to evaluate the behavior of systems under various scenarios, distinct from probabilistic sensitivity analysis which specifically assesses uncertainty’s impact on decision models.

####  Population

 The PICCOTEAM RC recommends identifying health and healthcare gaps to pinpoint all possible target populations of the early PM. (1) In earlier stages of early EE, conceive a diverse range of populations and corresponding settings for identifying use cases, which should be initially broad and later shortlisted based on technology development, external environment changes, and early stakeholder dialogues, informing the developer which populations to target for evidence generation. (2) Once the PM is seeking adoption or approved by a regulator, the evidence base of early EE should be limited to the relevant populations, informing payers which single or combination of results to use and whether to use general- or risk-stratified targeted populations like the blanket adoption of a multi-indication test (eg, circulating cell-free DNA for cancer early detection).^51^

####  Intervention

 PM’s intervention scope may change during technology development, which affects its positioning, product profiles, and value proposition. For instance, a diagnostic test demands higher sensitivity compared to a screening test, which may affect the positioning of a developing PM. (1) The intervention scope, initially exploratory for early EE, should explore all possible clinical pathways including the necessity of cascade testing (eg, via scenario analysis^52^). Though not finalized early on, researchers should eliminate dominated or infeasible options during technology development. (2) Considerations also include healthcare system readiness (eg, capacity, resources, and capabilities of a health system to effectively and efficiently deliver PM) and public perception through quantitative and/or qualitative approach during PM development.(3)New options may be added with technological breakthroughs or external shifts. (4) Middle-term and long-term plans of the medical innovator should be considered when shaping the intervention, for example, the medical innovator plans to develop a series of linked PM technologies.

####  Comparator

 (1) The comparator should be both applicable to clinical practice and appropriate for demonstrating the new technology’s value for money. (2) Involve key stakeholders, including medical innovators, clinicians, patients, and decision-makers, in the decision-making process to ensure the relevance and acceptance of the chosen comparator(s). (3) The choice of comparator depends on the specific characteristics of the new technology under consideration. This includes current practice, gold standard, patient preference, and competing technologies, covering both PM and non-PM technologies. (4) Emerging technologies should be considered by accounting for on-going trials and changes in PM’s pricing structures.^53^

####  Costs and Outcomes

 (1) Follow the PICCOTEAM RC for conventional EE to define (and prioritize) a checklist of to-be-measured costs, outcomes and potential value informing early developmental phase outcomes, including both expected direct impact of the developing PM and costs related to implementation, training, and potential downstream effects on healthcare systems. (2) In the absence of data, consider surrogate outcomes, standard expert elicitation^30^ and/or threshold analyses to determine cost-effectiveness of PM at various price-performance combinations. (3) Factor in additional outcomes like incidental findings and harm. (4) Update cost information across developmental stages and explore varying budget impacts of PM packages on family and society. For instance, technology costs might decrease while manpower costs may rise over time. (5) To determine the true cost of intervention, considering development, manufacturing plan, and marketing strategies from developers and manufacturers’ perspectives and/or final price from a payer’s perspective. (6) Gather input from medical innovators to explore various scenarios. (7) Additionally, access relevant societal values when feasible.^54^

####  Time Horizon

 Time horizon should not be affected by the technological development stage. The ideal case would be to consider lifetime and/or intergenerational impacts. Alternatively, a more pragmatic approach is to use a shorter timeframe to inform imminent and short-term cost-effectiveness, particularly for fast-evolving PM technologies. The choice of time horizon in early EEs should be tailored to diverse decision-makers’ perspectives and the specific contexts of PM technology.

####  Equity and Ethics

 The RC recommends early evaluation of equity and ethical concerns for effective PM technology and policy design, adopting a multidisciplinary approach involving researchers, clinicians, ethicists, policy-makers, and patient advocates. Discuss with stakeholders regularly to navigate the ethical and responsible development of PM. In particular, evaluate the influences of conducting EE and/or value of information analyses on the equity and ethical aspects of the developing PM technology.

####  Adaptability

 Engage in early regular discussions with regulatory agencies, clinicians, patients, payers, and other relevant stakeholders to learn their perspectives and needs to shape the HTA process, ensure alignment with regulatory standards and reimbursement policies over time.

####  Modelling

 (1) Develop a formative, explorative and adaptable model for Early EE of PM, ensure it accommodates diverse application types (eg, intended to be used for screening and/or diagnosis, in clinical setting or at home), potential clinical pathways and factors like cascade testing and harm. (2) At very early stages, conduct scenario and sensitivity analyses to assess PM’s value for money across different clinical pathways and guide product development, potentially using value of information analysis at a “later” early stage to inform more focused research. (3) Emphasize uncertainty analysis to identify robust intervention strategies, like varying risk and value thresholds or considering all parameter uncertainty in probabilistic analyses. (4) Foster iterative analyses via continuous interaction among researchers, stakeholders, and medical innovators. Use explorative modelling and value of information analysis^49,50^ to address uncertainties and identify robust policies.^55^

## Discussion

 For the first time, the PICCOTEAM RC provides a systematic framework for consistent analysis and reporting of PM EEs, ensuring quality, transparency, and adaptability of cost-effectiveness estimates across PM applications while addressing ethical and equity concerns. The RC is built on a value assessment framework that is deliberative and evidence-informed^56^ and incorporated best practices from the ISPOR taskforce and Health Technology Assessment International on deliberative processes.^16^ Unlike generic checklists such as Consolidated Health Economic Evaluation Reporting Standards,^17^ which emphasize transparent reporting and reproducibility but lack detailed methodological guidance especially for complex evaluations and Early EEs, the PICCOTEAM RC offers comprehensive guidance uniquely tailored to PM on planning, conducting, reporting, and appraising both Early and Conventional EEs. It is designed to accommodate diverse decision-making contexts across various PM types, addressing specific evaluation needs and complex methodological challenges at different developmental stages. Moreover, its structured guidance for evaluating PM ensures methodological consistency and improves the transferability of findings, particularly in LMICs. By reducing the need for independent assessments in resource-limited settings, it optimizes resource use and promotes equitable access to high-quality evaluations. As such, it can help address existing evaluation gaps identified in our previous review, promote credible and transferable evidence, and ultimately reduce inequities in access and decision-making. However, depending on the study’s location and design, users may additionally use region-specific and study-specific RCs, such as the International Decision Support Initiative RC for HTA in LMICs^11^ and the Indian RC for EE in India.^57^

 The HTA of PM bears unique methodological and policy challenges beyond the scope of traditional comparative effectiveness evidence-based medicine.^13^ In a 2022 methodology review of PM EEs, Bouttell and colleagues identified seven common challenges and recommended adapting existing methods for generic or diagnostic technologies to tackle them. However, they emphasized the need for new approaches to handle PM-specific challenges, including heterogeneity of tests and platforms, increased stratification that demands real-world evidence, incidental findings, and spillover effects. They also recommended leveraging early EE to facilitate process change for transparent regulation and adoption of PM.^58^ Following this, our methodology reviews underscore current methods that fall short in addressing PM’s unique healthcare paradigms, including clinical decision space of comprehensive testing, evolving test-treatment “hybrid,” and insufficient early EE guidance for PM. As an extension to existing methods, the PICCOTEAM RC addresses these concerns by integrating tailored recommendations to various PM applications across developmental stages, emphasizing expert engagement in a formative, iterative and progressive process of study design, data preparation and modelling, as well as unbiased extrapolation of real-world evidence and estimation of intergenerational effects. Furthermore, the RC provides detailed guidance on PM’s early-stage evaluation to mitigate uncertainties and potential biases, optimizing its development and positioning. Finally, this RC provides detailed recommendations to address PM-related LMICs and ethical issues.

 The PICCOTEAM RC has several limitations. First, this RC was formulated based on a comprehensive review of published studies mostly conducted in high-income countries of Europe, North America and Western Pacific,^12,13^ and refined by experts from Singapore, Thailand, Australia, United Kingdom, and the Netherlands. As such, researchers in other countries, particularly LMICs, may face additional challenges which might not be fully addressed in the PICCOTEAM RC. Second, while the PICCOTEAM RC has not been applied in real-world scenarios, plans are underway to conduct workshop trainings and pilot tests focused on to-be-introduced PM applications across various LMICs. Notably, the RC is currently being piloted in Thailand to evaluate screening and treatment strategies for FH, with findings to be published separately. Third, as Early EEs are not yet standard practice, the feasibility of PICCOTEAM RC’s recommendations involves addressing potential barriers, including collective efforts from manufacturers and policy-makers to create a conductive environment to adopt Early EEs into PM. Finally, it is crucial to view the PICCOTEAM RC as a “living document” reflecting the evolving scope and complexity of PM. For instance, future studies should continuously develop and update this RC to maintain its relevance and applicability in both LMICs and high-income countries.

## Conclusions

 In conclusion, the PICCOTEAM RC is a significant step towards standardizing and advancing PM EE, serving to improve the comparability, adaptability, and quality of PM’s estimated value for money. This RC can help decision-makers better understand and interpret study findings. However, it is important to acknowledge the RC’s limitations, promote its use in academic, industry, and public health authorities, and consistently refine it to adapt to PM’s evolving landscape. These proactive measures are essential to fully realize the potential of the PICCOTEAM RC in enhancing the regulation, reimbursement, and access to PM interventions in relevant populations.

## Ethical issues

 This study analyzed secondary data from published sources and presented a beta-version of the reference case for discussion in a stakeholder workshop. Participants were consulted in their professional capacity. The study did not collect nor report personal or sensitive information. In accordance with *IJHPM* policy, ethical approval was therefore not required.

## Conflicts of interest

 Authors declare that they have no conflicts of interest.

## Supplementary files


Supplementary file 1 contains a full list of the investigators of the Working Group.
