# Supplementary material for: Development of the PICCOTEAM Reference Case for Economic Evaluation of Precision Medicine
Source: Int J Health Policy Manag. 2025 Sep 8;14:8756. doi: 10.34172/ijhpm.8756 (PMC12595566; doi:10.34172/ijhpm.8756)
Supplement: Supplementary file 1 — contains a full list of the investigators of the Working Group. [file ijhpm-14-8756-s001.pdf]

Article title: Development of the PICCOTEAM Reference Case for Economic Evaluation of Precision Medicine

Journal name: International Journal of Health Policy and Management (IJHPM)

Authors' information: Wenjia Chen[1]*, Dimple Butani[2], Yi Wang[1], Janet Bouttell[3,4], Yah Ru Juang[1], Paul Scuffham[5,6], Janneke P.C. Grutters[7], Alec Morton[1,8,9], Hwee-Lin Wee[1,10], Joanne Ngeow[11,12], Vorasuk Shotelersuk[13,14], Yue Zhang[1], Laura Huey Mien Lim[1], Yot Teerawattananon[1,2], on behalf of the Working Group[#]

[1]Saw Swee Hock School of Public Health, National University of Singapore, Singapore, Singapore.

[2]Health Intervention and Technology Assessment Program (HITAP), Ministry of Public Health, Bangkok, Thailand.

[3]Centre for Healthcare Equipment and Technology Adoption, Nottingham University Hospitals NHS Trust, City Hospital, Nottingham, UK.

[4]Health Economics and Health Technology Assessment, School of Health and Wellbeing, University of Glasgow, Glasgow, UK.

[5]Centre for Applied Health Economics, School of Medicine and Dentistry, Griffith University, Nathan, QLD, Australia.

[6]Menzies Health Institute Queensland, Griffith University, Nathan, QLD, Australia.

[7]Science Department IQ Health, Radboud University Medical Center, Nijmegen, The Netherlands.

[8]Department of Management Science, University of Strathclyde Business School, Glasgow, UK.

[9]Duke-NUS Medical School, Singapore, Singapore.

[10]Department of Pharmacy, Faculty of Science, National University of Singapore, Singapore, Singapore.

[11]Cancer Genetics Service, Division of Medical Oncology, National Cancer Centre Singapore, Singapore, Singapore.

[12]Lee Kong Chian School of Medicine, Nanyang Technological University, Singapore, Singapore.

[13]Excellence Center for Genomics and Precision Medicine, King Chulalongkorn Memorial Hospital, the Thai Red Cross Society, Bangkok, Thailand.

[14]Center of Excellence for Medical Genomics, Department of Pediatrics, Faculty of Medicine, Chulalongkorn University, Bangkok, Thailand.

A full list of Collaborators of the Working Group is provided in Supplementary file 1.

***Correspondence to:** Wenjia Chen; Email: wenjiach@nus.edu.sg

Citation: Chen W, Butani D, Wang Y, et al. Development of the PICCOTEAM reference case for economic evaluation of precision medicine. Int J Health Policy Manag. 2025;14:8756. doi:10.34172/ijhpm.8756

Supplementary file 1

Working Group

Jingmei Li, PhD[1,2], Jing Lou, PhD[3], Sharon Li Ting Pek, PhD[4], Kar-Hui Ng, MD,PhD[5,6], Gandhi Naline, PhD[7], Briones Jamaica, PhD[3], Tasnim Sara, MSc[8], Peh Joo Ho, PhD[1,2,3], Zhou Huijun Brendon[9], PhD, Wanrudee Isaranuwatcha, PhD[10], Kingkaew Pritaporn, PhD[10], Rattanavipapong Waranya, BSc[10], Thamonwan Dulsamphan, BSc[10], Parntip Juntama, BSc[10], Chotika Suwanpanich, BSc[10], Nattiya Kapol, PhD[11], Namfon Sribundit PhD[11], Janewit Wongboonsin MD[12], Jate Ratanachina, MD[13,14,15], Bhoom Suktitipat, MD, PhD[16,17]

Affiliations

[1] Genome Institute of Singapore (GIS), Agency for Science, Technology and Research (A*STAR), 60 Biopolis Street, Genome, Singapore 138672, Singapore.

[2] Department of Surgery, Yong Loo Lin School of Medicine, National University of Singapore and National University Health System, Singapore, Singapore.

[3] Saw Swee Hock School of Public Health, National University of Singapore, 12 Science Drive 2, Singapore, 117549.

[4] Clinical Research Unit, Khoo Teck Puat Hospital, 768828, Singapore.

[5] Paediatrics, Yong Loo Lin School of Medicine, National University of Singapore, Singapore, Singapore.

[6] Shaw-NKF-NUH Children's Kidney Centre, Khoo Teck Puat-National University Children's Medical Institute, National University Hospital, NUHS Tower Block Level 12, 1E Kent Ridge Road, Singapore, 119228, Singapore.

[7] Duke-NUS Medical School, 8 College Rd, Singapore 169857.

[8] Lee Kong Chian School of Medicine, Nanyang Technological University, 50 Nanyang Avenue, Singapore 639798

[9] Precision Health Research, Singapore (PRECISE), 23 Rochester Park, #06-01, Singapore 139234.

[10] Health Intervention and Technology Assessment Program (HITAP), Ministry of Public Health, Bangkok, Thailand.

[11] Faculty of Pharmacy, Silpakorn University. Nakorn Pathom, Thailand.

[12] Renal Division, Brigham and Women's Hospital, Boston, Massachusetts, USA.

[13] National Heart and Lung Institute, Imperial College London, London, UK.

[14] Dept of Preventive and Social Medicine, King Chulalongkorn Memorial Hospital, The Thai Red Cross Society, Bangkok, Thailand.
[15] Dept of Preventive and Social Medicine, Faculty of Medicine, Chulalongkorn University, Bangkok, Thailand.
[16] Department of Biochemistry, Faculty of Medicine Siriraj Hospital, Mahidol University, Bangkok, Thailand.
[17] Integrative Computational BioScience (ICBS) Center, Mahidol University, Bangkok, Thailand.
